# Acute Bilateral Lower Limb Ischemia in a Middle-Aged Woman: Don’t Let the Obvious Distract You From the Occult

**DOI:** 10.7759/cureus.114818

**Published:** 2026-08-19

**Authors:** Mouza Alneyadi, Amani Alabdouli, Aisha Alyamahi, Thiagarajan Jaiganesh

**Affiliations:** 1 Emergency, Sheikh Tahnoon Bin Mohammed Medical City (STMC)/Tawam Hospital, Al Ain, ARE

**Keywords:** acute lower limb ischemia, atypical presentation, early recognition, multidisciplinary care, severe mitral stenosis, vascular emergency

## Abstract

Acute bilateral lower limb ischemia is a rare and life-threatening vascular emergency. Atypical presentations may lead to misdiagnosis, particularly when behavioral disturbances dominate the clinical picture. This report discusses a case of a 40-year-old South-Asian female who presented with severe bilateral lower limb pain and agitation, initially suspected to be psychiatric in origin. Further assessment revealed that the patient is suffering from acute bilateral lower limb ischemia. This case highlights the importance of thorough physical examination, maintaining a broad differential diagnosis, and early use of diagnostic imaging in atypical presentations of acute limb ischemia.

## Introduction

Acute lower limb ischemia (ALI) is a vascular emergency caused by a sudden reduction in arterial perfusion that threatens limb viability and requires urgent diagnosis and revascularization [1]. While ALI most commonly presents unilaterally, bilateral ALI is rare and usually reflects a proximal embolic source or systemic thrombotic process, carrying a higher risk of limb loss and mortality [1,2]. Common etiologies include cardiac embolism, acute aortic occlusion, aortic dissection, hypercoagulable states, and malignancy-associated thrombosis [2]. Diagnosis may be challenging because ALI can mimic conditions such as deep vein thrombosis, acute lumbar radiculopathy, compartment syndrome, cellulitis, or psychiatric disorders. Failure to perform an early vascular examination, including pulse assessment and bedside Doppler evaluation, is a common diagnostic pitfall that can delay treatment [1,2]. This case report describes an atypical presentation of bilateral ALI that was initially suspected to be of psychiatric origin. It highlights the importance of maintaining a high index of suspicion, performing an early vascular assessment, and using advanced imaging and multidisciplinary management to achieve timely diagnosis and treatment.

## Case presentation

A 40-year-old South-Asian female with no known past medical history presented to the emergency department (ED) with severe bilateral lower limb pain and agitation. She appeared highly distressed and uncooperative, limiting initial clinical assessment. Her vital signs on arrival were heart rate 90 bpm, respiratory rate 30 breaths/min, blood pressure 127/89 mmHg, and temperature 36.8°C. Physical examination demonstrated absent distal pulses in both lower limbs with cold extremities, and no Doppler signals were detected. Despite being in severe pain, the patient was moving both lower limbs spontaneously. No obvious pallor was appreciated, although assessment was limited by the patient’s darker skin tone. Moreover, a complete neurological examination of the lower limbs was limited by the patient’s severe agitation and poor cooperation. Two 50-mcg doses of intravenous fentanyl were administered for analgesia, and an interpreter was subsequently used to facilitate further history-taking.

**Figure 1:**
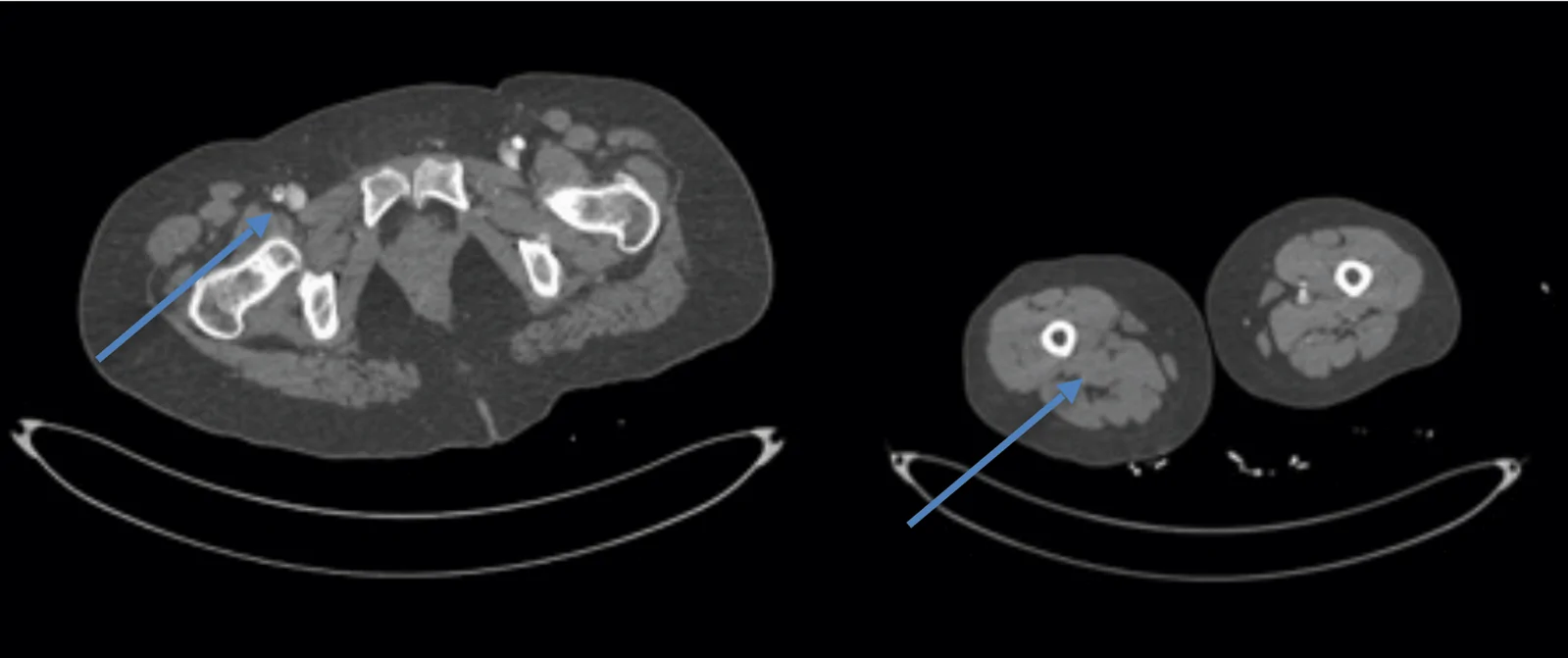
CT angiography of the lower limbs demonstrating bilateral acute limb ischemia Axial and coronal images showing long-segment occlusions of the right superficial femoral, popliteal, anterior tibial, and posterior tibial arteries (arrows), with markedly reduced distal perfusion bilaterally.

Venous blood gas analysis revealed metabolic acidosis with an elevated lactate level of 7.4 mmol/L, as shown in Table 1. CT angiography (Figure 1) demonstrated multiple arterial occlusions with poor bilateral lower limb perfusion. The patient received 5,000 units of intravenous heparin and was immediately transferred to the operating room for bilateral femoral artery embolectomy. Postoperatively, she was admitted to the critical care unit for four days and continued on a heparin infusion. She subsequently underwent bilateral fasciotomies for compartment syndrome secondary to rhabdomyolysis. A transthoracic echocardiogram later revealed severe mitral stenosis due to rheumatic heart disease, which was considered the likely embolic source. The patient was discharged on warfarin with follow-up arranged with vascular surgery and cardiology.

**Table 1:** Lab test results demonstrating high lactate and creatinine kinase levels

Test	Result	Reference Range
Sodium	139 mmol/L	135–145 mmol/L
Potassium	3.8 mmol/L	3.5–5.0 mmol/L
Creatinine	127 μmol/L	45–90 μmol/L (female)
Lactate	7.4 mmol/L	0.5–2.2 mmol/L
CK	>22,000 IU/L	26–192 IU/L

## Discussion

​This case report highlights the critical importance of maintaining a broad differential diagnosis and not overlooking severe underlying conditions due to atypical presentations. Initially, the patient's uncooperative behavior and distress led to a preliminary impression of a psychiatric disorder. However, thorough observation and a detailed physical examination revealed significant vascular compromise, which guided the correct diagnosis and treatment.

Bilateral lower limb ischemia is a rare and emergent condition, typically presenting with acute pain, pallor, pulselessness, paresthesia, paralysis, and poikilothermia (the six Ps) [2,3]. However, our patient’s initial presentation was dominated by restlessness and screaming, which are not classic symptoms of vascular occlusion. Although pain is the hallmark symptom of ALI, severe agitation may represent an atypical manifestation of advanced ischemia [4]. Intense ischemic pain, together with metabolic derangements resulting from tissue hypoperfusion, including lactic acidosis and ongoing muscle ischemia, can produce marked restlessness, confusion, and uncooperative behavior [4,5]. In severe cases, agitation may be mistaken for a primary psychiatric disorder, as occurred in our patient, or for delirium, potentially delaying recognition of this limb-threatening condition. Therefore, this case emphasizes that unexplained agitation in a patient with acute lower limb pain should prompt an immediate vascular assessment, including examination of peripheral pulses and bedside Doppler evaluation, to avoid diagnostic delay and facilitate timely revascularization [5].

Simultaneous bilateral ALI due to systemic embolism is exceptionally rare and is reported mainly as isolated case reports. Most published cases are associated with cardiac embolic sources, including atrial fibrillation, left ventricular thrombus, cardiac myxoma, or rheumatic mitral stenosis [1]. In contrast to many previously reported cases, our patient initially presented predominantly with agitation rather than classic ischemic findings, which initially obscured the diagnosis. The combination of bilateral multilevel embolic occlusion, atypical behavioral presentation, and previously undiagnosed severe rheumatic mitral stenosis makes this case rare and provides important clinical learning points.

A key learning point from this case is the importance of not dismissing significant findings despite an initial misleading presentation. The absence of peripheral pulses and Doppler signals in the lower extremities was pivotal in shifting the focus from a psychiatric to a vascular etiology. These findings prompted immediate vascular consultation and advanced imaging, which confirmed the diagnosis of multiple arterial occlusions.

The management of this case also highlights the role of multidisciplinary teamwork in the ED. The collaboration between emergency medicine, vascular surgery, and cardiology was essential in providing timely and effective care. The use of bedside ultrasonography and prompt CT angiography facilitated rapid diagnosis and appropriate surgical intervention, which is crucial in preventing irreversible tissue damage and loss of limb function [6].

Furthermore, this case emphasizes the importance of pain management and patient communication in the acute setting. The administration of fentanyl helped alleviate the patient’s pain, allowing for a more comprehensive assessment. The involvement of an interpreter was also crucial in obtaining a more accurate history, which further guided the clinical management [7].

Another critical aspect of this case is the identification of an underlying chronic condition - severe mitral stenosis due to rheumatic heart disease that had not been previously diagnosed. This finding underscores the importance of a thorough systemic evaluation in patients presenting with unexplained acute arterial embolism, as rheumatic mitral stenosis is a well-recognized source of systemic embolization [8].

## Conclusions

In conclusion, early recognition and prompt management of acute bilateral lower limb ischemia are essential to prevent permanent disability and life-threatening complications. This case emphasizes the value of maintaining a broad differential diagnosis, especially in the presence of atypical clinical presentations, and highlights the crucial role of thorough physical assessment and multidisciplinary care in improving patient outcomes.

## References

[1] Björck M, Earnshaw JJ, Acosta S (2020). Editor's choice - European Society for Vascular Surgery (ESVS) 2020 Clinical Practice Guidelines on the management of acute limb ischaemia. Eur J Vasc Endovasc Surg.

[2] Creager MA, Kaufman JA, Conte MS (2012). Clinical practice. Acute limb ischemia. N Engl J Med.

[3] O'Connell JB, Quiñones-Baldrich WJ (2009). Proper evaluation and management of acute embolic versus thrombotic limb ischemia. Semin Vasc Surg.

[4] Simon F, Oberhuber A, Floros N, Busch A, Wagenhäuser MU, Schelzig H, Duran M (2018). Acute limb ischemia-much more than just a lack of oxygen. Int J Mol Sci.

[5] Santistevan JR (2017). Acute limb ischemia: an emergency medicine approach. Emerg Med Clin North Am.

[6] Venkatesan A, Halai M, Choke E, Enoch S, Nichol A (2020). Acute limb ischaemia. BMJ.

[7] Karliner LS, Jacobs EA, Chen AH, Mutha S (2007). Do professional interpreters improve clinical care for patients with limited English proficiency? A systematic review of the literature. Health Serv Res.

[8] Carapetis JR, Steer AC, Mulholland EK, Weber M (2005). The global burden of group A streptococcal diseases. Lancet Infect Dis.

